# Research advances in plant–insect molecular interaction

**DOI:** 10.12688/f1000research.21502.1

**Published:** 2020-03-19

**Authors:** Chun-Yu Chen, Ying-Bo Mao

**Affiliations:** 1 Chinese Academy of Sciences (CAS) Key Laboratory of Insect Developmental and Evolutionary Biology, CAS Center for Excellence in Molecular Plant Sciences, Shanghai Institute of Plant Physiology and Ecology, University of CAS, Chinese Academy of Sciences, Shanghai, China; 2 National Key Laboratory of Plant Molecular Genetics, CAS Center for Excellence in Molecular Plant Sciences, Shanghai Institute of Plant Physiology and Ecology, University of CAS, Chinese Academy of Sciences, Shanghai, China

**Keywords:** Plant defense, Insect herbivory, Jasmonate (JA), Elicitor, Effector

## Abstract

Acute and precise signal perception and transduction are essential for plant defense against insects. Insect elicitors—that is, the biologically active molecules from insects’ oral secretion (which contains regurgitant and saliva), frass, ovipositional fluids, and the endosymbionts—are recognized by plants and subsequently induce a local or systematic defense response. On the other hand, insects secrete various types of effectors to interfere with plant defense at multiple levels for better adaptation. Jasmonate is a main regulator involved in plant defense against insects and integrates with multiple pathways to make up the intricate defense network. Jasmonate signaling is strictly regulated in plants to avoid the hypersensitive defense response and seems to be vulnerable to assault by insect effectors at the same time. Here, we summarize recently identified elicitors, effectors, and their target proteins in plants and discuss their underlying molecular mechanisms.

## Introduction

There are about 1 million insects and over 300,000 plants on our planet, and plant–insect interactions are the driving force of biodiversity. With long-term co-evolution, plants and insects have developed sophisticated mechanisms for adaptation ^1^. In general, plants can recognize herbivore-/damage-/microbe-associated molecular patterns (HAMPs/DAMPs/MAMPs) and make the right defense. The early defense responses contain depolarization of the plasma transmembrane potential, changes of cytosolic Ca ^2+^, reactive oxygen species (ROS) burst, and mitogen-activated protein kinase (MAPK) ^2, 3^. Most of these reactions are able to activate jasmonate (JA)-mediated plant defense ^4, 5^. JA is a main regulator of plant defense and its synthesis and regulation have been extensively studied ^6– 9^. Recent studies reveal new insights in JA oxidative metabolism and their negative regulation in the JA pathway ^10, 11^. In most plants, JA-Ile is the active signal recognized by the COI1 and promotes JAZ–COI1 interaction leading to JAZ degradation. This relieves the JAZ-interacting transcription factors to activate downstream defense gene expressions ^12– 16^. However, in *Marchantia polymorpha*, MpCOI1 recognized OPDA-Ile instead of JA-Ile. That work revealed the ligand-receptor co-evolution of the JA signaling pathway in land plants ^17^. MYC2 is a well-studied transcription factor in JA signaling and can interact with both JAZ and MED25, the subunit of the mediator complex. The JAZ proteins recruit TOPLESS scaffold protein to inhibit gene transcription, whereas MED25 brings COI1 to MYC2 targeting promoters ^18^. In this model, COI1 is thought to be the nuclear receptor. JAT1, which localizes at the nuclear envelope and plasma membrane, is the transporter responsible for the influx of JA-Ile into nucleus ^19^. To balance the tradeoff between growth and defense, plants strictly regulate JA signaling to avoid a hypersensitive defense response ^20, 21^. Some development regulators, including SPLs and DELLAs, target JAZ or MYC transcription factors to modulate JA signaling output ^22– 26^. Interestingly, some insects use similar strategies to attenuate plant defense for fitness.

Herbivorous insects have different mouthparts and feeding habits. Active molecules from insects’ oral secretion (OS) (which contains regurgitant and saliva), frass, ovipositional fluids, and the endosymbionts of insects have a large impact on plant defense. Some of these molecules used by plants to trigger specialized defense are called elicitors, and those to weaken the plant defense response are defined as effectors. Plant–insect recognition is the first and also the key step of an effective defense in plants ^27, 28^. In this review, we discuss recent research advances in insect elicitors and effectors and their roles in plant–insect interactions.

## Plant perceptions of insect herbivory

**Figure 1.**
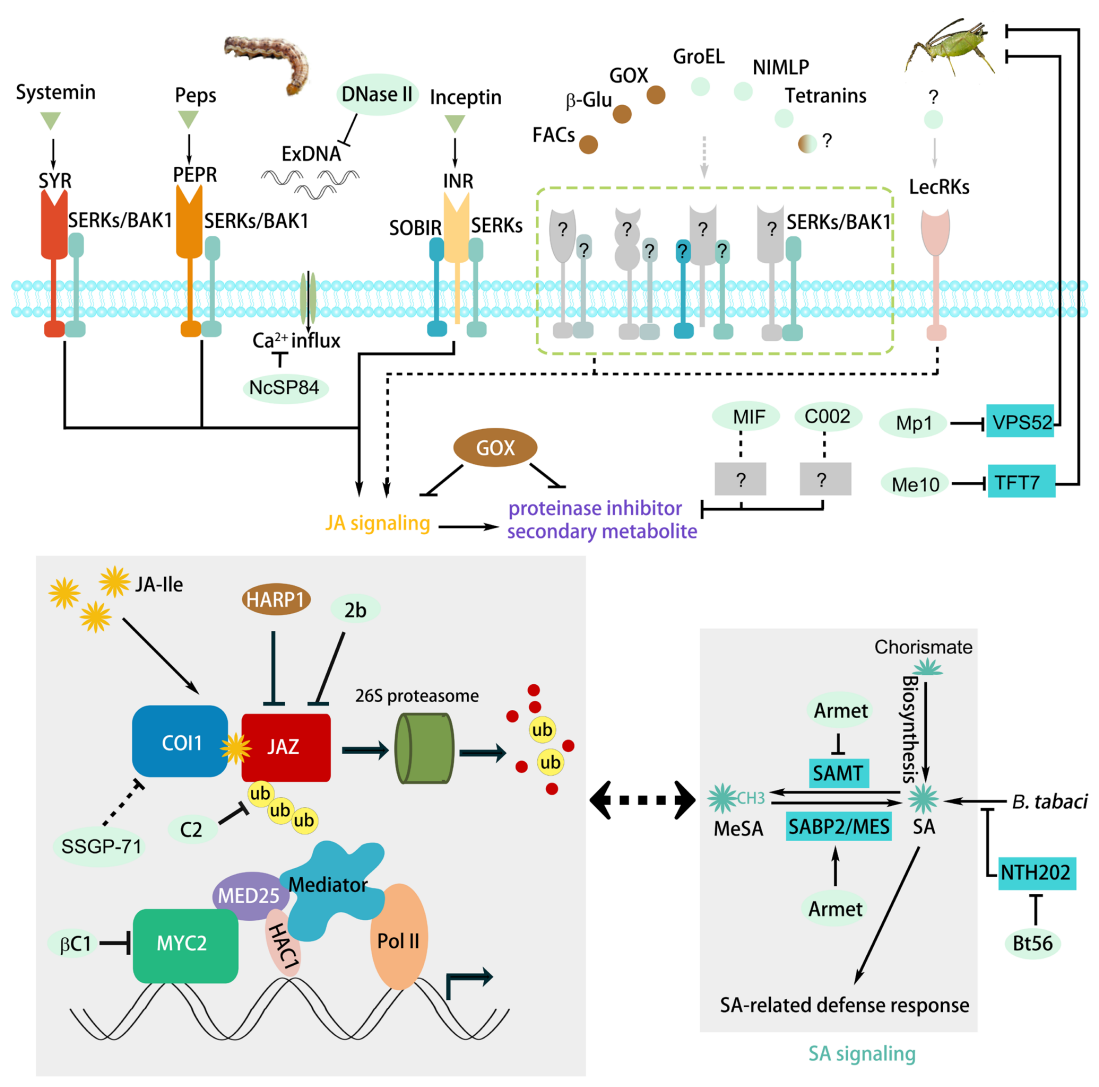
Schematic diagram of herbivory-associated elicitors and effectors manipulating plant defense. Receptors (SYR, PEPR, INR, and LecRKs) located on the plant cell surface recognize small peptides (sytemine, inceptin, and Peps) and, together with the co-receptors (SERKs/BAK1 and SOBIR1), trigger downstream defense signaling. Also, elicitors derived from insects, including FACs, β-Glu, and GOX, are able to activate plant defense with the unknown mechanisms. On the other hand, insects secrete effectors to weaken plant defenses. Some effectors interfere with jasmonate (JA) signaling directly (HARP1, 2b, C2, βC1, and SSGP-71) or indirectly (Armet and Bt56) by enhancing salicylic acid (SA) accumulation to compromise JA signaling. Some effectors (Mp1 and Me10) target plant proteins (VPS52 and TFT7) that are directly involved in defense. The DNase II eliminates the extracellular DNA which is released by damaged cells to trigger plant defense. MIF and C002 from aphids are of benefit to insects living on the host plants, but the underlying mechanisms remain elusive. Notably, some elicitors/effectors are plant-specific. Here, the GOX from *Helicoverpa zea* acts as an effector, inhibiting nicotine accumulation in tobacco, and, on the other hand, acts as an elicitor specifically inducing plant response in tomato.

Plant perception of an insect attack is the first step of defense. Insect herbivory raised diverse active molecules such as damage-associated molecules, insect-derived elicitors, and the plant endogenous molecules activated by insect digestive enzymes ( Figure 1). The specific and efficient recognition of these active molecules guarantees the timely priming of plant defense ^29, 30^.

### Plant-derived signal molecules activated by herbivory

Wounding damage caused by insect herbivory will quickly trigger plant defense signaling. The first reported damage-related peptide signal was systemin, an 18–amino acid polypeptide cleaved from prosystemin (inactive form) in tomato upon wounding stimulus ^31^. Systemin promotes JA accumulation and activates the expression of genes encoding proteinase inhibitors which have insecticide activity ^32^. Whereas systemin had been reported long before, its receptor SYR1, a leucine-rich repeat receptor kinase (LRR-RK), was identified recently. The introgression line, which lacks SYR1 expression, is highly sensitive to *Spodoptera littoralis* ^33^. Besides systemin, other wound-induced peptides had been identified in plants, including *Arabidopsis*, maize, and rice. The application of synthetic 23–amino acid maize Peps could mimic the *Spodoptera exigua* attack, and similar Peps were found in rice recently ^34, 35^. In *Arabidopsis*, AtPeps, which is generated from PROPEPs under the catalyzation of the cysteine protease METACASPASE4 (MC4), acts as signals to trigger both JA and SA signaling pathways ^36, 37^. Like the systemin-SYR1 module, the reported receptors of AtPeps—AtPEPR1 and AtPEPR2—are also classified in the LRR-RK family ^38, 39^.

From *Spodoptera frugiperda* larval OSs, researchers isolated a disulfide-bridged peptide (+ICDINGVCVDA−), termed inceptin, that can induce the accumulation of defense hormones such as ethylene, JA, and SA in cowpea plants. Inceptin is the proteolytic fragment of chloroplastic ATP synthase γ-subunit of cowpea plants digested by *S. frugiperda* larvae ^40, 41^. Recently, on BioRxiv, it was reported that the receptor of inceptin in plants was a leucine-rich repeat receptor-like protein, INR, which is distinguished from LRR-RKs by lacking an intracellular kinase domain ^42^. These findings expand the paradigm of plant surface recognition of insect herbivory.

### Elicitors secreted by insects

Besides plant signal molecules activated by insect feeding, a number of reported elicitors are derived from insects themselves and most of them belong to HAMPs ^43^. It had been reported that the OS, the oviposition and the honeydew of insects could induce a plant defense response, including the accumulation of JA and secondary metabolism ^44^. These insect-derived elicitors can be classified as fatty acid derivatives, enzymes, and some other proteins ^43^.

The first identified fatty acid–amino acid conjugate (FAC) elicitor was volicitin, which was isolated from *S. exigua* larval OSs. Volicitin can induce the emission of volatiles in maize to attract predators ^45^. After volicitin, other forms of FACs from various insect OSs had been found in succession ^46, 47^. In *Nicotiana attenuate*, FACs from *Manduca sexta* activate the MAPK pathway ^48^. Besides FACs, califerins, the sulfooxy fatty acids that exist in OSs of grasshopper ( *Schistocerca americana*) larvae, also have elicitor activity ^49^. Glucose oxidases (GOXs) and β-glucosidase are enzyme-like elicitors. GOX is identified from *Helicoverpa zea* and specifically activates defense response in tomato ^50, 51^. The β-glucosidase in *Pieris brassicae* larval OSs triggers the emission of volatiles from wounded cabbage leaves and this attracts predators such as parasitic wasp ^52, 53^. Lipase and phospholipase C are other types of salivary enzyme-like elicitors. Lipase of *Schistocerca gregaria* OS elevates oxylipin accumulation and defense response in *Arabidopsis* ^54^. Phospholipase C of *Spodoptera frugiperda* induces the accumulation of proteinase inhibitors in corn ^55^.

The above-mentioned elicitors are from chewing insects. The elicitors from the piercing-sucking insects are isolated largely from salivary glands. The mucin-like salivary protein (NlMLP) of planthopper ( *Nilaparvata lugens*) is a double-edged sword. On one hand, it contributes to the formation of salivary sheaths for successful feeding; on the other hand, it was used by plants to trigger a defense response, like Ca ^2+^ mobilization, the MEK2 MAPK cascades, and JA signaling transduction, thereby reducing the performance of *N. lugens* ^56^. Tetranins is another characterized elicitor identified from *Tetranychus urticae*. Tetranins increases the expression of defense genes and activates JA, salicylic acid (SA), and abscisic acid biosynthesis in plant. It also promotes volatile emission to attract predatory mites ^57^.

Some elicitors are from endosymbionts. MAMPs could be released through herbivory OSs and recognized by plants to induce pattern-triggered immunity (PTI) ^58, 59^. The chaperon GroEL from the endosymbionts *Buchnera* of potato aphids ( *Macrosiphum euphorbiae*) induces oxidative bursts and PTI in *Arabidopsis* ^60^. From the *S. littoralis* larval OSs, the porin-like proteins most likely of bacterial origin can induce the early response of plant defense ^61^. A recent report reveals that some elicitors are from honeydew-associated microbes in sucking arthropods ^62^.

## Insect effectors twist plant defense

To adapt to their host plants, insects have developed multilayered means for fitness. Besides releasing elicitors, the insect releases effectors that disturb host plant defense response for successful feeding ^63^. The reported insect effectors are identified from both the herbivory itself and insect-related microbiomes ( Figure 1).

The first reported insect effector was GOX from the chewing insect, *H. zea*, which inhibits nicotine accumulation and elevates the SA-mediated PR-1a protein level in tobacco ^64, 65^. Notably, the same GOX protein induces plant response in tomato ^50, 51^, which we discussed in the ‘Elicitors secreted by insects’ section. This suggests that the same protein acts as the effector or as the elicitor depending on their interacted host plant. Another piece of evidence in support of insect effectors is that the *S. littoralis* larvae that fed on OS pretreated plants had a greater weight increase ^66^.

The direct interaction with JA signaling-related components is an efficient way for herbivory effectors to inhibit plant defense. In our recent work, we isolated a venom-like protein termed HARP1, which is identified from the OS of *Helicoverpa armigera.* HARP1 can interact with multiple JAZ proteins of *Arabidopsis* and cotton plants to prevent COI1-mediated JAZ degradation, thereby blocking the JA signaling output ^67^. SSGP-71 is an E3 ubiquitin ligase–mimicking protein in Hessian fly ( *Mayetiola destructor*). It allows the insect to hijack the plant proteasome and block the basal immunity ^68^. These studies fill in the gap of the working mechanism about how insects manipulate effectors to block plant defense for better adaptation.

Some insect effectors inhibit plant defense by interfering with the crosstalk between SA and JA. For example, Bt56 from the whitefly ( *Bemisia tabaci*) enhanced the performance of the whitefly on tobacco by decreasing JA signaling through the antagonism between JA and SA. Bt56 could directly interact with KNOTTED 1-like homeobox transcription factor NTH202 and eliminate the negative modulation of NTH202 on SA accumulation ^69^. Armet, the effector of pea aphid ( *Acyrthosiphon pisum*) protein, induced SA accumulation by blocking SA methylation and enhanced the pathogen resistance in plants, reflecting a novel tripartite interaction of insect–plant–pathogen ^70, 71^.

The extracellular DNA and hydrogen peroxide that are released by damaged cells can trigger plant defense ^30^. Therefore, some insects secrete effectors to eliminate the production of these DAMPs. The planthopper ( *Laodelphax striatellus*) secretes salivary DNase II, which acts as an effector by erasing extracellular DNA, and the *Trichoplusia ni* salivary catalase functions as an ROS scavenger to reduce hydrogen peroxide, thus inhibiting ROS burst and other plant defense responses ^72, 73^.

Moreover, some effectors were reported to target other defense-related proteins in plants. A set of saliva proteins in aphids were proven to have effector activity through proteomic combined RNA sequencing (RNA-seq) analysis ^63, 74– 79^. A macrophage migration inhibitory factor (MIF) from pea aphid saliva inhibits immune response in *N. benthamiana* and improves aphid performance. Interestingly, the MIFs in vertebrates are also involved in the immune pathway, suggesting the highly conserved function of MIF ^80, 81^. Vacuolar protein sorting-associated protein 52 (VPS52) in potato ( *Solanum tuberosum*) has negative impacts on green peach aphid ( *Myzus persicae*) infection. *M. persicae* saliva-secreted protein Mp1 targets the VPS52 as an effective virulence strategy ^77, 82^. Me10 from *M. euphorbiae* interacts with tomato TFT7, a 14-3-3 isoform involved in aphid resistance, and enhances aphid longevity and fecundity ^83^. Some effectors can target the host cell wall. Expansin-like protein (HaEXPB2) from the nematode ( *Heterodera avenae*) binds to cellulose of tobacco, thereby increasing nematode infectivity ^84^.

The effectors mentioned above are generated from the insect itself. Other effectors are also derived from insect-borne microbe. Although the exact effector components need to be explored, it was found that Colorado potato beetle ( *Leptinotarsa decemlineata*) larvae suppress tomato defense response by exploiting bacteria in their OSs and gut ^85, 86^. Besides bacteria, some active molecules from vector-borne pathogens are reported to interfere with plant defense and are of benefit for their insect vectors living on host plants ^87^. The phytoplasm protein SAP11 and SAP54 of aster yellows phytoplasma strain witches’ broom was proposed to promote aphid colonization and also interfere with plant development ^88– 90^. The βC1 of tomato yellow leaf curl China virus directly interacts with MYC2 protein to decrease the MYC2-regulated terpene synthase, thereby reducing plant resistance to the whitefly ^91^. The 2b protein of the aphid-borne cucumber mosaic virus (CMV) stabilizes JAZ proteins by direct interaction, thus blocking JA signaling output, and this benefits aphid ( *M. persicae*) performance on the host plant ^92^. The C2 protein of tomato yellow leaf curl virus can also compromise JA signaling in tobacco by interacting with plant ubiquitin to block JAZ1 protein degradation, thereby reducing plant resistance to the insect vector whitefly ^93^. These studies reveal the intricate interaction of plant–virus–insect vector. In Table 1, we summarize the reported insect-associated elicitors and effectors from different species and their probable roles.

**Table 1.** Herbivory-associated elicitors and effectors.

		Name	Origin	Biofunction	References
Elicitors	Plant-derived	Systemin	Wounded tomato plants	Perceived by SYR1, induce accumulation of proteinase inhibitor and ethylene, and induce oxidative bursts	31, 33
		PEPs	Wounded plants ( *Arabidopsis*, maize, rice)	Induce defensin and burst of hydrogen peroxide **(**H _2_O _2_) after perceiving by PEPRs	36, 38, 39
		Inceptin	Degradation of cowpea ATP synthase by *Spodoptera* *frugiperda* during herbivory	Increase the concentration of JA and SA by interacting with INR	40, 42
	Derived from insect	Volicitin	*Spodoptera exigua*	Induce volatiles emission in corn	45
		Caeliferins	*Schistocerca americana*		49
		GOX	*Helicoverpa zea, Ostrinia nubilalis*	Specifically promote defense response in tomato	50, 51
		β-glucosidase	*Pieris brassicae*	Increase volatile emission in cabbage	52
		Lipase	*Schistocerca gregaria*	Elevate the oxylipins accumulation in *Arabidopsis*	54
		Phospholipase C	*S. frugiperda*	Trigger proteinase inhibitors accumulation in corn	55
		Bruchins	*Bruchus pisorum*	Induce neoplasms formation beneath the insect egg in pea	94, 95
		NlMLP	*Nilaparvata lugens*	Induce plant defense response in rice	56
		Tetranins	*Tetranychus urticae*	Cytosolic calcium influx and membrane depolarization induce biosynthesis of JA. SA and ABA in kidney bean	57
		GroEL	*Buchnera* in *Macrosiphum* *euphorbiae*	Induce PTI and ROS accumulation in *Arabidopsis*	60
		Porin-like proteins	*Bacteria in Spodoptera littoralis*	Trigger membrane potential changes and cytosolic Ca2 ^+^ elevations in *Arabidopsis* and *Vicia faba*	61
		Unidentified	Gut-associated bacteria in *H. zea*	Increase salivary GOX to induce defense in tomato	96, 97
		Unidentified	Honeydew-associated microbes *N. lugens*	Induce accumulation of phytoalexins and volatile emission in rice	62, 98
Effectors	Insect-derived	GOX	*H. zea*	Decrease nicotine accumulation in tobacco	64
		HARP1	*Helicoverpa armigera*	Interact with and stabilize JAZs, depress JA signaling in *Arabidopsis*	67
		SSGP-71	*Mayetiola destructor*	Interact with Skp, decrease plant proteasome activity, thus block hormone signaling in wheat	68
		Bt56	*Bemisia tabaci*	Interact with NTH202 to increase SA biosynthesis, thus decrease JA response in tobacco	69
		Armet	*Acyrthosiphon pisum*	Help feeding of insect, induce SA accumulation and pathogen response in *N. benthamiana* and *Medicago truncatula*	70, 71
		DNase II	*Laodelphax striatellus*	Erase extracellular DNA released by damaged cell in rice	72
		Catalase	*Trichoplusia ni*	Reduce H _2_O _2_ in tomato	73
		C002	*A. pisum*, *Myzus persicae*	ApC002 and MpC002 help insect foraging and feeding on fava bean and *N. benthamiana*, respectively	63, 74
		MIF	*A. pisum*, *M. persicae*	Improve aphid performance, inhibit immune response in *N. benthamiana*	80
		Mp1	*M. persicae*	Interact with VPS52 to relocalize to vesicle-like structures and enhance insect virulence in *Arabidopsis* and potato	82
		Me10	*M. euphorbiae*	Interact with TFT7, enhance the longevity and fecundity on tomato	83
		Mp42, Mp55 Me23	*M. persicae*, *M. euphorbiae*	Increase aphid reproduction, suppress *N. benthamiana* defenses	99, 100
		HaEXPB2	*Heterodera avenae*	Bind to cellulous and target cell wall when parasitizing *N. benthamiana*	84
		Phosphatase 2C	*M. destructor*	Interfere with the wheat signal transduction pathway possibly by phosphatase ability	101
		Endo-beta-1,4- Glucanase (NIEG1)	*N. lugens*	Degrade celluloses in plant cell wall, enable insect stylet to reach the rice phloem	102
		NcSP75	*Nephotettix cincticeps*	Help successful ingestion from sieve elements of rice	103
		NcSP84	*N. cincticeps*	Suppress accumulation of Ca2 ^+^ and H _2_O _2_ and sieve element clogging in rice	104
		NlSEF1	*N. lugens*	Help successful ingestion from sieve elements of rice	105
	Derived from insect-borne microbe	Unidentified	Gut and oral secretion–associated bacteria in Colorado potato beetle	Suppress tomato defense response Bind and destabilize TCPs, reduce plant defense in *Arabidopsis*	85, 86
		SAP11	Aster yellows witches’ broom in *Macrosteles quadrilineatus*		106
		SAP54	Aster yellows witches’ broom in *M.* *quadrilineatus*	Degrade MTFs through interacting with RAD23, influence floral development in *Arabidopsis*	90
		βC1	Tomato yellow leaf curl China virus in *B. tabaci*	Interact with MYC2 and suppress MYC2-regulated terpene synthesis in *Arabidopsis*	91
		2b	Cucumber mosaic virus (CMV) in *M. persicae*	Interact with and stable JAZ protein, blocking JA signaling in *Arabidopsis*	92
		C2	Tomato yellow leaf curl virus in *B.* *tabaci*	Interact with plant ubiquitin, blocking JA signaling in tobacco	93

## Prospects

JA is a conserved defense regulator in the plant kingdom. On one hand, various elicitors can be recognized by plants to trigger JA signaling. On the other hand, the JA pathway tends to be targeted by a diverse range of attackers for fitness ( Figure 1). Some insect effectors have a mechanism similar to that of the virus proteins in blocking JA signaling ^67, 91– 93^. It would be interesting to study whether there are relationships between the phylogeny of insect effectors and viral proteins. Although numerous elicitors and effectors were identified, their target proteins, the underlying mechanisms, and the transportation mechanisms of the effectors entering plant cells are largely unknown and deserve further investigation. In plants, JA is integrated with multiple signaling to form a complex and flexible defense network. Recent research has revealed the intricate defense network shaped by insect herbivory ^69, 107– 109^. Studies have also shown that insects can use plant defense metabolites to find their host plants and to fend off predators ^110, 111^; this gives new insight into plant–insect interactions. Further investigations will greatly enrich our knowledge of the complex and flexible interactions between plants and insects and will also be helpful for breeding insect-proof crops ^112, 113^.
